# Discovery of a Non-Nucleoside SETD2 Methyltransferase Inhibitor against Acute Myeloid Leukemia

**DOI:** 10.3390/ijms221810055

**Published:** 2021-09-17

**Authors:** Dávid Bajusz, Zsolt Bognár, Jessica Ebner, Florian Grebien, György M. Keserű

**Affiliations:** 1 Medicinal Chemistry Research Group, Research Centre for Natural Sciences, 1117 Budapest, Hungary; bajusz.david@ttk.hu (D.B.); zsolt.bognar@epfl.ch (Z.B.); 2 Institute for Medical Biochemistry, University of Veterinary Medicine, 1220 Vienna, Austria; Jessica.Ebner@vetmeduni.ac.at (J.E.); Florian.Grebien@vetmeduni.ac.at (F.G.)

**Keywords:** histone methyltransferase, AML, SETD2 inhibitor, virtual screening

## Abstract

Histone methyltransferases (HMTs) have attracted considerable attention as potential targets for pharmaceutical intervention in various malignant diseases. These enzymes are known for introducing methyl marks at specific locations of histone proteins, creating a complex system that regulates epigenetic control of gene expression and cell differentiation. Here, we describe the identification of first-generation cell-permeable non-nucleoside type inhibitors of SETD2, the only mammalian HMT that is able to tri-methylate the K36 residue of histone H3. By generating the epigenetic mark H3K36me3, SETD2 is involved in the progression of acute myeloid leukemia. We developed a structure-based virtual screening protocol that was first validated in retrospective studies. Next, prospective screening was performed on a large library of commercially available compounds. Experimental validation of 22 virtual hits led to the discovery of three compounds that showed dose-dependent inhibition of the enzymatic activity of SETD2. Compound C13 effectively blocked the proliferation of two acute myeloid leukemia (AML) cell lines with MLL rearrangements and led to decreased H3K36me3 levels, prioritizing this chemotype as a viable chemical starting point for drug discovery projects.

## 1. Introduction

In eukaryotic cells, histone proteins play instrumental roles in the packaging of DNA inside the nucleus. The four major histones (H2a, H2b, H3, and H4) form a disc-shaped octameric complex, providing a structural framework for binding of the DNA double helix to form chromatin [1]. In addition to this role, the side chains of histone proteins are subject to highly specific post-translational modifications, including methylation, acetylation, and others. These functional groups are added to histone tails by specific enzymes and serve as epigenetic marks, which participate in the regulation of gene expression. The presence or absence of distinct histone modifications can effect a change between the relaxed (euchromatin) and condensed (heterochromatin) states of chromatin [1]. In mammalian cells, SET domain containing 2 (SETD2) is the only known lysine methyltransferase (KMT) that is able to tri-methylate the K36 lysine side chain of the H3 protein, resulting in the H3K36me3 epigenetic mark [2]. As most other KMT proteins, SETD2 uses S-adenosyl methionine (SAM) as a cofactor and source of electrophilic methyl groups. By binding to SETD2, the ε-amino group of the H3K36 lysine residue comes in close proximity to the carbon atom of the methylsulfonium cation, which has a partial positive charge in the active site, enabling an S_N_2-type nucleophilic attack [3].

The H3K36me3 mark was implicated in various processes, including transcriptional elongation, alternative splicing, and DNA repair [4]. Inappropriate expression of SETD2 and resulting errors in H3K36 trimethylation have been linked to various types of malignancies [5], for example human breast cancer [6], clear cell renal cell carcinoma (cRCC) [7], and systemic mastocytosis (SM) [8]. Furthermore, SETD2 is highly expressed in leukemia [9]. In relapsed pediatric acute lymphoblastic leukemia (ALL) patients, SETD2 mutations were found at an increased frequency, highlighting a possible role of SETD2 in chemotherapy resistance [10]. In line with that, the heterozygous loss of SETD2 caused resistance to DNA-damaging agents in cell lines and mouse models of leukemia [11].

**Figure 1:**
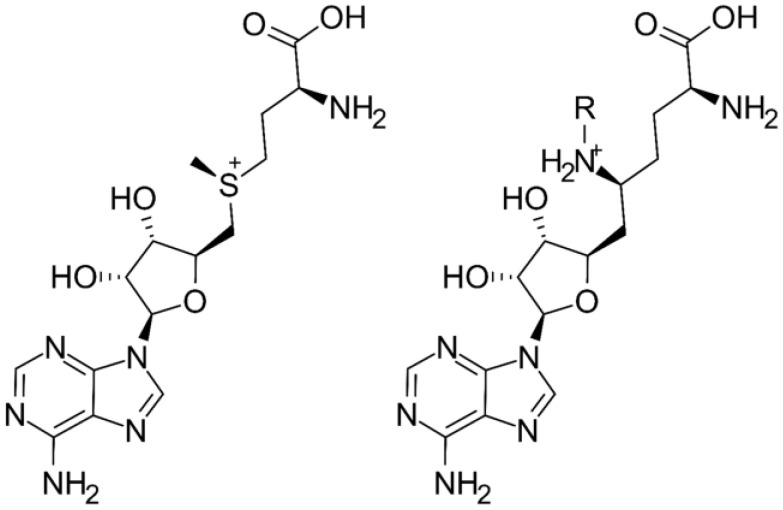
The 2D structures of S-adenosyl-methionine (SAM) (left) and sinefungin (SNF) (right). Substitution at the R group yields sinefungin analog SETD2 inhibitors with micromolar potencies (in the parent compound, the amine is unsubstituted).

We recently reported that the expression and activity of SETD2 is crucial for the progression of acute myeloid leukemia (AML) with MLL rearrangements. The downregulation of SETD2 inhibited the development and progression of MLL-rearranged AML in vitro and in vivo and led to a reduction in both H3K36me3 and H3K79me2 levels. Additionally, loss of SETD2 induced hypersensitivity against the DOT1L inhibitor pinometostat (EPZ-5676; currently in phase 1 and 2 clinical trials, see NCT03701295 and NCT03724084 at https://clinicaltrials.gov, accessed on 1 August 2021) in MLL-rearranged AML cells [12]. Currently, the only known small-molecule inhibitors of SETD2 are sinefungin and its synthetic nucleoside analogues. Sinefungin (SNF) is a naturally occurring compound isolated from bacteria of the *Streptomyces* genus [13], which was described as a non-selective inhibitor of various SET-domain containing protein lysine methyltransferases (PKMTs) [14]. Notably, sinefungin is a close analog of S-adenosyl-methionine (having a primary amine group in place of the S-methyl group, Figure 1). A series of sinefungin analogs, in which the amine group was extended with apolar sidechains, was reported to inhibit SETD2 at a low micromolar concentration in vitro [3,15] (these are summarized in the Supplementary Materials, Section S1), with the N-isobutyl derivative being the most potent with an IC_50_ value of 0.29 µM. 

The clinical relevance of SETD2 in cancer, along with the lack of further inhibitor chemotypes and the general ADME/bioavailability concerns around nucleoside analogs (such as sinefungin), prompted us to launch a virtual screening campaign with the aim of identifying small molecule inhibitors of SETD2 with novel chemical scaffolds. Such compounds could fulfill a scientific and clinical need by serving as both chemical probes for target validation and target engagement studies, and as initial hits for a drug discovery campaign. The SETD2 X-ray structures with sinefungin analogs bound to the SAM binding site provided ample structural data for our efforts to develop a virtual screening protocol. To avoid restricting the explored chemical space, while keeping the computational demands at a manageable scale, knowledge-based pre-screening steps (substructure filtering and pharmacophore screening) were introduced to the workflow prior to ligand docking. This approach identified three new compounds with experimentally confirmed SETD2 inhibitory activities, one of which also effectively reduced H3K36me3 levels and blocked the proliferation of the SETD2-dependent AML cell lines. The compound C13 contains a non-nucleoside core scaffold and can be considered a potential starting point for optimized SETD2 inhibitors.

## 2. Results

### 2.1. Virtual Screening Workflow and Retrospective Validation

**Figure 2:**
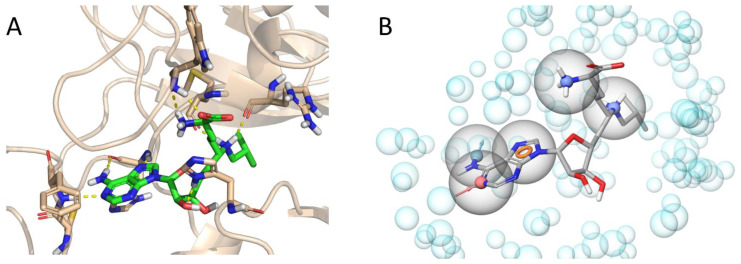
(**A**) Isobutyl-sinefungin (iBu-SNF, PDB:5LSY [15]) in the SAM-binding pocket of SETD2. (**B**) Consensus pharmacophore model overlaid on the structure of iBu-SNF.

To identify the new chemotypes of SETD2 inhibitors, we set out to conduct a structure-based virtual screening campaign on the Mcule database [16], with nearly six million commercially available compounds, using ligand docking. To avoid the high computational demand of docking all these compounds, we introduced pharmacophore screening as a computationally less expensive pre-filtering step into the workflow. This was implemented based on the structural and protein–ligand interaction characteristics of the existing SETD2 inhibitors, i.e., sinefungin analogs. Examining the X-ray structure of isobutyl-sinefungin (the most potent SETD2 inhibitor with an IC_50_ value of 0.29 µM, Figure 2A), we observed that in addition to the H-bond network that anchors the adenine core, further H-bonds and cation–π interactions are provided by the ornithine unit and the charged secondary amine (and for SAM, the methylsulfonium cation) to stabilize the position of the apolar sidechain toward the hydrophobic channel of the enzyme. On the level of pharmacophores, this translates to a particularly rich set of pharmacophoric features (Figure S1). From these, a consensus pharmacophore model was derived and applied during the pre-screening step (Figure 2B). The pharmacophore model contained the key interacting features of the adenine core, as well as two positively charged groups and was capable of retrieving the nine known SETD2 inhibitors from our training set (nine known inhibitors with 450 decoy compounds), with a minimal number of false positives (Supplementary Materials, Section S2). To note, the pharmacophore model boasts an excellent performance, as expressed by receiver operating characteristic (ROC) enrichments of 40 and 28 at 1% and 2% false positive rates, respectively [17]; an area under the ROC curve (AUC) value of 0.97 and a Boltzmann-Enhanced Discrimination of ROC (BEDROC) value of 0.702.

For docking, eight PDB (Protein Data Bank) structures were initially considered and two of them (5JLE [18] and 5LSY [15]) were finally selected based on testing a large number of configurations in terms of the protein structures and docking constraints to be used (Supplementary Materials, Section S3). The docking protocol retrieved all nine known inhibitors from the above-mentioned training set with excellent early enrichment factors (size-independent ROC enrichment factors of 80 and 44 at 1% and 2% false positive rate, respectively [17]) and an area under the ROC curve (AUC) value of 0.97, which was further increased to 0.99 by the application of pharmacophore-based pre-screening; BEDROC values were 0.816 and 0.854, respectively.

### 2.2. Prospective Screening and Hit Selection

Next, we set out to apply the assembled virtual screening workflow to identify new SETD2 inhibitors from a large supplier database (Mcule, ca. 6 million compounds) in a prospective fashion. While pharmacophore screening presents a more economical alternative to docking in terms of the computational demand, it requires the generation of a conformational ensemble for each molecule prior to screening. For 5–6 millions of compounds, this would generate data in the terabyte range. To reduce this, we introduced a permissive substructure filter as an additional pre-screening step. It utilized a generic query substructure that enabled us to keep any molecule with at least a minimal structural resemblance to the natural cofactor SAM and sinefungin derivatives, i.e., those that contain an aromatic core and an amine group, separated by a linker region of at least five atoms (Supplementary Materials, Section S4).

**Figure 3:**
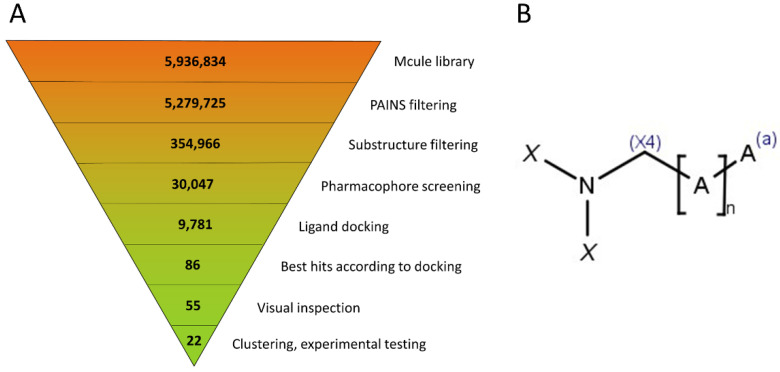
(**A**) Virtual screening workflow, with the number of compounds at each step indicated. (**B**) Generic substructure filter used for pre-screening. A symbolizes any non-hydrogen atom, (a) means that the atom is in an aromatic bond, and (X4) implies an sp^3^ hybridization state. X is either H or C(X4), allowing for (but not requiring) an arbitrary sidechain. The linker length is defined as 4 ≤ *n* ≤ 10 (this allows for linear or ring-containing linkers as well).

The virtual screening was carried out in a stepwise fashion; the exact number of compounds at each step is summarized in Figure 3. The starting database of about 6 million compounds was cut down first by about 0.65 million by excluding the compounds with reactive groups or Pan Assay Interference Compounds (PAINS) [19]. Next, the permissive substructure filter retained ca. 355,000 compounds, of which 30,047 were able to form at least one conformational state that fit to the requirements of the pharmacophore filter. These were docked into the appropriate receptor grids with Glide Standard Precision (SP) docking, resulting in 9781 compounds that were successfully docked into at least one of the receptor grids.

To select compounds for purchasing and experimental testing, we considered two criteria: in addition to the 50 compounds with the best docking scores, 50 other compounds were kept based on the fact that they exhibited similar binding poses to the three most potent inhibitors (based on their Tanimoto similarities to a consensus interaction fingerprint [20]). Due to overlaps, this resulted in 86 unique compounds, whose docking poses were visually inspected in detail, checking for the main interacting features such as an adenine-mimicking core and a positively charged amine at the active center. The resulting 55 ligands were clustered and 22 representative compounds were chosen as virtual hits and purchased for experimental testing.

### 2.3. Experimental Hit Confirmation and Characterization

The 22 purchased virtual hits were first assayed in vitro to evaluate their inhibitory effect in a chemiluminescence-based SETD2 enzyme inhibition assay (for details, see Supplementary Materials, Section S5 and Figure S4A). In this assay, the SETD2-specific H3K36 tri-methylation is measured with an anti-H3K36me3 antibody and a recombinant SETD2 protein. As a control, we used sinefungin, which fully inhibited the enzymatic activity of SETD2 at a concentration of 2 mM (Figure S4B). The compounds were primarily screened in singlets at a concentration of 100 µM (Figure S4B). In a second round, 17 compounds were retested in duplicates, identifying 12 compounds that inhibited the enzymatic function of SETD2 by at least 50% (Figure S4C). These 12 preferred compounds were selected for IC_50_ measurements in duplicates.

**Figure 4:**
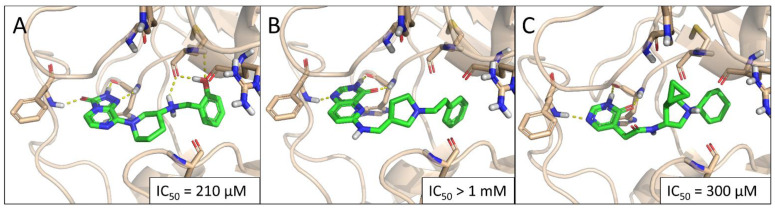
Predicted binding poses of the hit compounds C13 (**A**), C17 (**B**), and C19 (**C**).

The three virtual hits C13, C17, and C19 inhibited SETD2 activity in a dose-dependent manner, with IC_50_ values in the high micromolar range. For compound C17, 50% inhibition was not reached within its window of solubility (1 mM assay concentration). Predicted binding poses for these hits are summarized in Figure 4 (IC_50_ curves are included in Figure S5). Notably, the three compounds contain three different core scaffolds, and based on their predicted binding poses, all three are strongly anchored to the adenine binding pocket by three alternating H bonds (acceptor-donor-acceptor motif), nominating them as new SETD2 inhibitor scaffolds that can be utilized for inhibitor design.

Finally, we aimed to assess the inhibitory effects of the three confirmed hits in cells. We used MOLM-13 and MV4-11 cells, which represent two AML cell lines harboring MLL rearrangements together with the FLT3-ITD mutations, as we previously demonstrated that those cell lines are sensitive to SETD2 perturbation [12].

**Figure 5:**
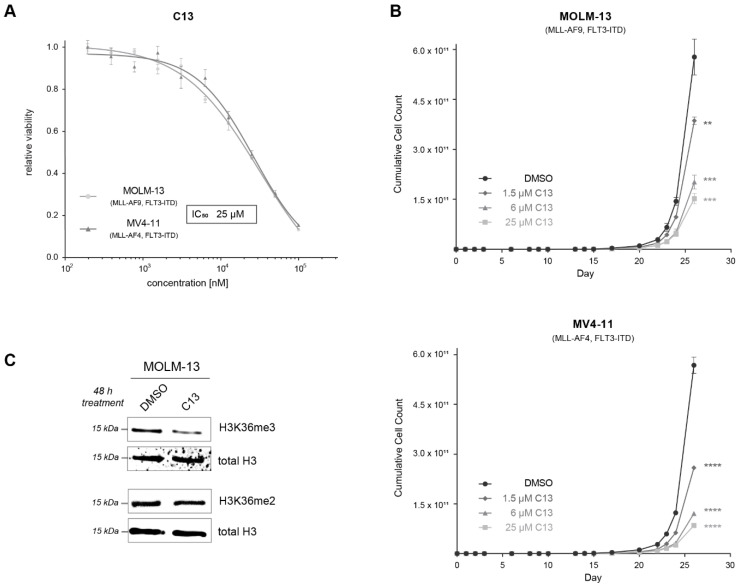
The confirmed hit compound C13 inhibits the proliferation of leukemia cell lines and inhibits the enzymatic activity of SETD2 in cells. (**A**) Dose–response curves of MOLM-13 and MV4-11 cells treated for 5 days with different concentrations of C13, resulting in an IC_50_ value of 25 µM. (**B**) Proliferation assay of AML cell lines treated with three different concentrations of C13 and DMSO as control. Cells were treated every 48 or 72 h, and cell counts were determined in regular intervals; IC_10_ = 1.5 µM, IC_20_ = 6 µM, IC_50_ = 25 µM. (**C**) Western blot analysis of MOLM-13 cells treated for 48 h with 100 µM C13 or DMSO. Membranes were incubated with antibodies against H3K36me3 and H3K36me2. Total H3 was used as a loading control. ** *p* < 0.01, *** *p* < 0.001, **** *p* < 0.0001.

Interestingly, only C13 induced a dose-dependent anti-proliferative effect in the two cell lines (Figure 5A, IC_50_ = 25 µM), thus nominating it as our primary hit compound. The lack of cellular efficiency found for C17 and C19 suggests that these chemotypes need further physicochemical optimization to achieve appropriate solubility and permeability.

Next, we aimed to further characterize the potential of C13 to inhibit SETD2-driven cell proliferation. Three different concentrations of C13 (IC_10_ = 1.5 µM, IC_20_ = 6 µM, and IC_50_ = 25 µM) inhibited the proliferation of both MOLM-13 and MV4-11 cell lines in these long-term culture experiments (Figure 5B). Finally, we evaluated whether C13 is able to inhibit SETD2 activity in cells. We treated MOLM-13 cells for 48 h with 100 µM C13 and determined H3K36me3 levels via Western blotting (Figure 5C). We found that C13 treatment induced a specific downregulation of H3K36me3 levels, while H3K36me2 levels remained unaffected. This result confirms the target engagement of C13 inhibiting the KMT activity of SETD2 in a cellular context [21].

Meanwhile, we noticed that the observed SETD2 inhibitory activity of C13 (IC_50_ = 210 µM) is weaker than its cellular potency (IC_50_ = 25 µM), which hints at the possibility that C13 exerts its cellular effect through the inhibition of more histone methyltransferases, similar in structure to SETD2. Although we did not have access to further methyltransferase assays, we examined this possibility by a brief docking study against the available PDB structures of 15 other methyltransferases (Supplementary Materials, Section S7). This analysis has highlighted six histone methyltransferases: NSD1, ASH1L, SETMAR, EHMT2, SUV420H1, and SMYD2, as further potential targets.

In summary, our validation experiments indicate that C13 is a non-nucleoside SETD2 inhibitor with reasonable cellular potency that nominates this chemotype for further optimization as a new treatment option for acute myeloid leukemia.

## 3. Discussion

Our recent findings have provided ample evidence for the role of SETD2 in the progression of acute myeloid leukemia (AML) with MLL rearrangements [12]. Since there were limited research efforts for targeting SETD2 so far, the only currently known small-molecule SETD2 inhibitors are sinefungin and its N-alkylated synthetic analogues [15]. In agreement with the hydrophobic character of the histone-binding channel of SETD2, it was observed that a hydroxyl group in the alkyl chain drastically decreased the inhibitory activity. However, as sinefungin is not cell-permeable, it cannot be used for cellular studies. It is worth noting that SETD2 inhibition also generated interest in the pharma sector, since Epizyme (now part of Genentech) reported a potent SETD2 inhibitor with a yet-undisclosed structure [22].

Given the relevance of SETD2 in cancer, we set out to identify new non-nucleoside SETD2 inhibitor compounds by virtual screening. To that end, we assembled a computationally efficient, stepwise screening protocol that employs multiple concepts and validated it against the available set of known SETD2 inhibitors. After using the protocol to prospectively screen a large supplier database, we tested 22 virtual hits, three of which inhibited SETD2 in vitro in a concentration-dependent manner. Notably, the three confirmed hits contain three different, new, non-nucleoside SETD2 inhibitor scaffolds, which could be utilized for further medicinal chemistry efforts. The hits were further pursued to assess their inhibitory activities in the leukemia cell lines MOLM-13 and MV4-11. This revealed that the hit compound C13 effectively blocked SETD2-mediated proliferation of MLL-rearranged AML cell lines, thereby nominating it as the primary hit compound identified in this study. Furthermore, C13 induced the downregulation of H3K36me3 while leaving H3K36me2 levels unchanged, confirming SETD2 target engagement at the cellular level. We should note that the enzymatic inhibitory activity of C13 is weaker than we had hoped for, highlighting the need for further optimization to turn it into a potent lead compound. On the other hand, this is the first non-nucleoside inhibitor that is suitable for cellular studies as well (in contrast to the natural substrate that has limited cell permeability).

Altogether, compound C13 represents a promising starting point to develop specific inhibitors of SETD2. The availability of specific small-molecule inhibitors of SETD2 would serve as important chemical probes to investigate the role of SETD2 in cancer and other diseases.

## 4. Materials and Methods

### 4.1. Datasets and Ligand Preparation

The structures and activity data of these known SETD2 inhibitors were retrieved from primary literature mentioned above [3,15]. Fourteen X-ray structures of SETD2 with various ligands available from these publications were obtained from the Protein Data Bank (PDB). We used the Mcule database of purchasable, in-stock compounds, containing 5,936,834 entries [16]. These database operations were carried out using KNIME (KNIME AG, Zürich, Switzerland) [23] and Instant JChem (ChemAxon LLC: Budapest, Hungary) [24]. The compounds with reactive groups were removed with Schrödinger Ligfilter, while the Pan Assay Interference Compounds (PAINS) were removed with the PAINS filters of Canvas [19]. Prior to ligand preparation, the computationally less demanding substructure filtering (see Supplementary Materials, Section S4) was carried out, resulting in about 355,000 compounds to proceed with. These were prepared with Schrödinger LigPrep (Schrödinger, LLC: New York, NY, USA), generating 3D structures to possible tautomeric and protomeric states significantly populated in an aqueous medium at pH = 7.4 ± 1.5 [25,26]. As a necessity for the subsequent screening step, Schrödinger ConfGen (Schrödinger, LLC: New York, NY, USA) was used to generate 50 conformational states for each structure generated by the LigPrep [26,27].

### 4.2. Pharmacophore Screening

The E-pharmacophore module of Schrödinger’s Phase [28,29] was used to create a pharmacophore model for each of the five PDB structures (4FMU [3], 5LSS, 5LSX, 5LSY, and 5LT6 [15]), containing the most active (low micromolar or submicromolar) inhibitors of SETD2, namely Pr-SNF, iBu-SNF, Bn-SNF, and Pe-SNF, respectively, as ligands. From these, a consensus pharmacophore model was derived based on their performance to retrieve all nine known inhibitors from the training set consisting of: (i) the nine known inhibitors and (ii) a pool of 450 decoy compounds generated with the DUD-E server (see Supplementary Materials, Section S2 for more detail) [30].

### 4.3. Ligand Docking

The fourteen X-ray structures for SETD2 available in the PDB were prepared using the Schrödinger Prime One-step Protein Preparation interface with the default settings [25,26]. Briefly, the hydrogen atoms were added, and the most likely protonation states were calculated for ionizable side chains at a pH of 7.4 with the PROPKA plugin [31]. Those entries that contained a fragment of the H3 histone protein (5JJY, 5JLB, 5V21, and 5V22 [18]) were omitted (as none of these contained active inhibitors as ligands), while the remaining structures were superimposed. The 5LSX [15] structure was missing crucial amino acid residues in the binding pocket (1671–1673), and 4H12 [3] was in a closed conformation, making it inaccessible for the substrate; these files were also excluded from further study. Our aim was to identify representative structures from the remaining eight entries that, when combined, show the best performance in an ensemble docking scenario, reproducing the crystallographic docking poses of the original ligands with favorable docking scores and the lowest root mean square distance (RMSD) values. We also validated this protocol by performing docking on the training set described earlier, demonstrating a promising enrichment of active compounds. Finally, two of the remaining eight structures were chosen for prospective screening (for more details, see Supplementary Materials, Section S3): 5JLE [18] and 5LSY [15]. One of these structures, 5JLE, was published in a study investigating the peptide binding properties of SETD2. Interestingly, however, this specific structure does not contain a histone peptide, but it is a binary complex of SETD2 and SAH (S-adenosyl-homocysteine, the product of the enzyme catalyzed reaction). Similarly, 5LSY is a binary complex between SETD2 and N-isobutyl-sinefungin, the most potent sinefungin analog. Since both structures contain small molecules at their nucleoside binding site, they are considered suitable for virtual screening of these small molecule libraries to identify the new chemotypes bound to this site. The water molecules were removed from the binding site of both structures prior to docking. This way, ligands are allowed to displace or even replace binding-site waters that would add further gain in binding free energy. Schrödinger Glide was used for grid generation and docking (standard precision, SP) [26,32,33].

### 4.4. Post-Processing and Hit Selection

The successfully docked poses were evaluated by two measures: the Glide docking score as a crude approximation of the binding energy, and similarity to a consensus interaction fingerprint derived from the three most active inhibitors of SETD2 (5LSY, 5LSX, and 5LSS [15], featuring the ligands iBu-SNF, Bn-SNF, and nPr-SNF, respectively), as an assessment of the docking pose. At locations where the individual fingerprints of these structures did not match, the corresponding bit was set manually based on a visual inspection. A fingerprint was generated for each of the predicted docking poses, and the Tanimoto similarity [20] was calculated from the consensus IFP with KNIME [23]. For the docked ligands, a high similarity value indicates an interaction pattern with the receptor that is similar to that of the most active inhibitors. After a visual inspection, the remaining 55 compounds were clustered based on their molecular fingerprints (hashed, linear fingerprints in Schrödinger Canvas) [26,34] with the “average” linkage rule. The number of clusters was determined by the Kelley criterion, but the largest cluster was further divided into two subclusters based on the presence or absence of a distinctive substructure ((1S,5R)-3,6-diazabicyclo[3.2.2]nonane). A representative compound was selected from each cluster for purchasing and testing.

### 4.5. Measurement of SETD2 Enzyme Activity

The chemiluminescence assay used here assesses SETD2-specific tri-methylation activity toward lysine 36 at histone H3 (H3K36me3) in an in vitro setup (see Figure S4A for the assay principle). The measurement of SETD2 activity was performed according to the manufacturer’s protocol (Catalog-No. 52060, https://bpsbioscience.com/setd2-chemiluminescent-assay-kit-52060, accessed on 1 August 2021). These values were blank-corrected and normalized. The enzymatic activities were determined relative to the positive control of the kit (full enzymatic activity) and to the known SET domain inhibitor, sinefungin, which was used as a negative control (complete enzymatic activity inhibition). The compounds used were diluted in DMSO and used at 100 µM. The IC_50_ values were calculated from at least five concentrations in duplicates with serial dilutions starting from 1 mM, using the Prism8 software (GraphPad, San Diego, CA, USA).

### 4.6. Evaluation of Antiproliferative Effects of C13 in Leukemia Cell Lines

MOLM-13 and MV4-11 human leukemia cell lines were obtained from DSMZ (Deutsche Sammlung von Mikroorganismen und Zellkulturen GmbH, 2014, www.dsmz.de, accessed on 1 August 2021). MOLM-13 and MV4-11 leukemia cell lines were seeded in white 96-well plates at a density of 5 × 10^3^ cells/well and treated with C13 in biological triplicates at indicated concentrations. Five days after treatment, cell viability was evaluated using the CellTiter-Glo Luminescent Cell Viability Assay (Promega, Madison, WI, USA) on a SPARK multimode microplate reader (Tecan Trading AG, Männedorf, Switzerland). IC_50_, IC_20_, and IC_10_ values were calculated using Prism8 software (GraphPad, San Diego, CA, USA). Detailed information on experimental procedures for cell culture and Western blotting is included in the Supplementary Materials, Section S6.

## Data Availability

The data presented in this study are available in the article and Supplementary Materials. Source data for the virtual screening computations and biochemical measurements are available from the authors upon request.

## Supplementary Materials

The following are available online at https://www.mdpi.com/article/10.3390/ijms221810055/s1.
