# Supplementary material for: Discovery of a Non-Nucleoside SETD2 Methyltransferase Inhibitor against Acute Myeloid Leukemia
Source: Int J Mol Sci. 2021 Sep 17;22(18):10055. doi: 10.3390/ijms221810055 (PMC8471172; doi:10.3390/ijms221810055)
Supplement: Supplementary file 1 [file ijms-22-10055-s001.zip › ijms-1349019-supplementary.pdf]

Supplementary Material

Discovery of a non-nucleoside SETD2 methyltransferase inhibitor against acute myeloid leukemia

Zsolt Bognár[1,‡], Dávid Bajusz[1,‡], Jessica Ebner[2,‡], Florian Grebien[2], György M. Keserű[1,]*

[1] Medicinal Chemistry Research Group, Research Centre for Natural Sciences, Magyar tudósok krt. 2, 1117 Budapest, Hungary

[2] Institute for Medical Biochemistry, University of Veterinary Medicine, Veterinärplatz 1, 1220 Vienna, Austria

‡These authors contributed equally.

*Corresponding author: György M. Keserű, keseru.gyorgy@ttk.hu

Table of contents

1. Inhibitory activities of reported sinefungin analogs 2
2. Developing the pharmacophore model for pre-screening 2
3. Docking protocol 5
4. Substructure filter 8
5. Virtual hits and primary screening results 9
6. Cellular Validation of hit compound C13 19
7. Other potential targets 20
8. References 22

1. Inhibitory activities of reported sinefungin analogs

Table S1. IC_{50} values or (in lack thereof) percent inhibition values of reported sinefungin analogs.[1,2]

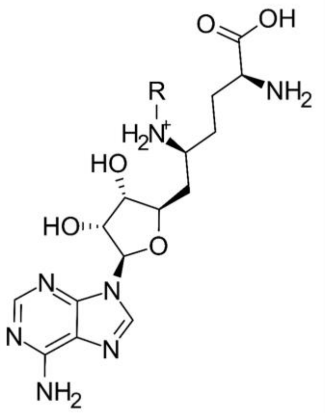

R group	IC_{50} (μM)[2]	IC_{50} (μM)[1]	% Inh. @ 30 μM[1]
H (sinefungin)	28 ± 2		71
Me	> 100		
Et	8 ± 1		
Pr	0.8 ± 0.2	0.49	
iBu		0.29	
nPent		3.9	
Bn	0.5 ± 0.1	1.2	
$CH_2CH(OH)Me$			46
$(CH_2)_3OH$			36

2. Developing the pharmacophore model for pre-screening

The E-pharmacophore module of Schrödinger's Phase[3,4] was used to create a pharmacophore model for each of five PDB structures (4FMU,[2] 5LSS, 5LSX, 5LSY, and 5LT6[1]) containing the most active (low micromolar or submicromolar) inhibitors of SETD2, namely Pr-SNF, iBu-SNF,

Bn-SNF, and Pe-SNF as ligands. The full set of pharmacophore features in N-alkyl sinefungin derivatives are illustrated in Figure S1.

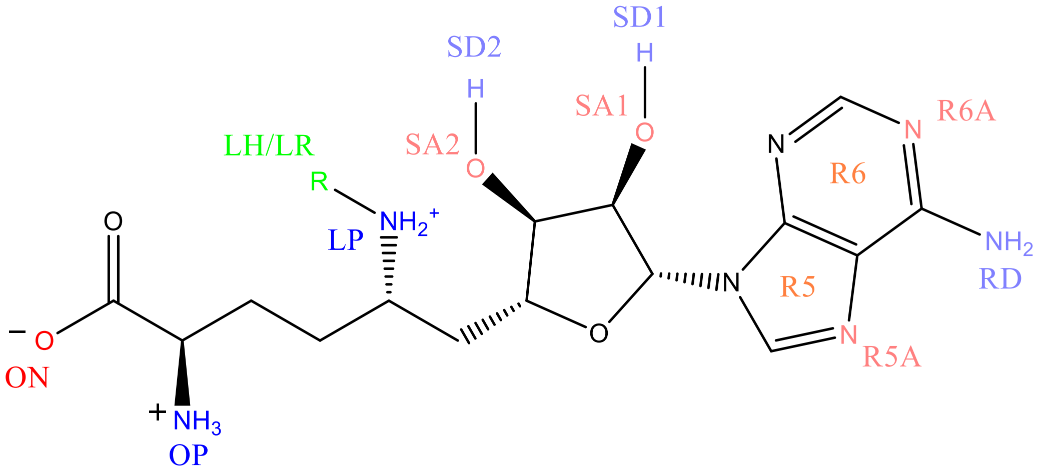

Figure S1. The pharmacophore features of N-alkyl sinefungins contain positively (LP, OP) and negatively charged (ON) groups, as well as H-bond acceptors (R5A, R6A, SA1, SA2) and donors (RD, SD1, SD2), aromatic rings (R5, R6) and depending on the R substituent, an extra aromatic ring (LR) or hydrophobic group (LH).

The E-pharmacophore module evaluates a protein-ligand complex by scoring the ligand in place with the Glide XP scoring function and breaking down the docking score into score contributions of the different pharmacophoric features to assess their importance in the formation of the protein-ligand complex. (A larger negative score contribution means an energetically more favorable interaction.) To construct a consensus model, the docking score contributions for each feature were determined in the models, these are summarized in Table S2.

Table S2. Glide XP docking score contributions of the pharmacophoric features of N-alkyl sinefungins.

Ligand	PDB	R6	R5	R6A	RD	R5A	SA1	SD1	SA2	SD2	OP	ON	LP	LH/LR
Pr-SNF	4fmu	0	-0.76	-1.7	-1.7	0	-2.04	0	0	-0.7	0	-0.7	0	0
Pr-SNF	5lss	-1.18	0	0	-1.7	-1.7	0	-1.6	-1.42	0	-1	0	-1	0
Bn-SNF	5lsx	0	-0.71	-2.2	-0.69	0	-0.16	0	0	-1.6	-1	0	-1	-2.03
iBu-SNF	5lsy	0	-0.55	-2.2	-0.7	0	0	0	0	-1.38	-1	0	-1	-0.3
Pe-SNF	5lt6	-1.21	0	0	-0.7	-2.2	0	0	-1.6	0	-0.96	0	-0.96	-0.6

Based on this data, the pharmacophore elements labeled R6A, RD, OP and LP were clearly significant, but R5 and SD2 should also be taken into further consideration. Pharmacophore model variations were established using different combinations of these elements, also evaluating whether accepting an equivalence between hydrogen bond donors and positive charges is advantageous or not. The appropriate elements were selected based on the 5LSS[1] PDB structure, with a tolerance range of 2.00 Å. The atoms of the receptor binding pocket were marked as exclusion zones, forcing the model to discard any ligands that would make a steric clash with the receptor. The training set was prepared as described in the Materials and methods section and was screened using each of these model variations. The results are summarized in Table S3. Here, the HYP3 pharmacophore model has minimized the number of retrieved decoys (false positives), while keeping all nine of the known SETD2 inhibitors, therefore this model was selected for further usage (its 3D representation is shown in Figure 2B). The HYP3 model boasts excellent early enrichment factors (ROC enrichments of 40 and 28 at 1% and 2% false positive rate, respectively[5]), an area under the ROC curve (AUC) value of 0.97, and a BEDROC value (at an α setting of 20.0) of 0.702.

Table S3. Pharmacophore model (hypothesis) variants, with the number of actives and decoys retrieved from the training set. (D=P means that hydrogen bond donors and positive charges were considered to be equivalent.)

Hypothesis	Pharmacophore elements							Actives found	Decoys found
HYP1	R6A	RD	OP	LP				8	38
HYP2	R6A	RD	OP	LP			D=P	9	340
HYP3	R6A	RD	OP	LP	R5			9	110
HYP4	R6A	RD	OP	LP	R5		D=P	9	401
HYP5	R6A	RD	OP	LP	R5	SD2		9	302
HYP6	R6A	RD	OP	LP	R5	SD2	D=P	9	428
HYP7	R6A	RD	OP	LP		SD2		9	149
HYP8	R6A	RD	OP	LP		SD2	D=P	9	395

3. Docking protocol

To determine which PDB structures to use for docking, we assessed their performances individually and in combination. Nine of the original ligands present in the available PDB structures (SAM, SAH, SNF, and various N-alkyl sinefungins) were prepared with LigPrep, as described in the Materials and methods section. Grid files were generated for each of the eight PDB structures of interest. Since the binding of SAM to SETD2 shows strong anchoring features (similarly to the binding mode of ATP to protein kinases), we considered using docking constraints. The characteristic hydrogen bonding pattern of the adenine core was mimicked by incorporating the backbone NH and carbonyl oxygen of His1629, as well as the backbone NH of Phe1679 as possible locations for hydrogen bond constraints. Three additional constraint sites were also examined, corresponding to the anchoring residues of the protonated secondary amine: Arg1625, Met1627, and Tyr1604. The ligands were redocked into the receptor grids using Schrödinger Glide standard precision (SP) docking,[6–8] optionally with different combinations of the aforementioned constraints.

Somewhat surprisingly, we were able to obtain the most promising docking scores and RMSD values by using none of the constraints, as summarized in Table S4. Nonetheless, to obtain more relevant docking poses during prospective screening, we have incorporated a post-docking check for the constraints, requiring at least two of the three H-bonds in the adenine pocket to be formed.

Table S4. A) Docking scores for each ligand with different constraint settings (best score out of the eight structures). B) RMSD values for each ligand with different constraint settings (best score out of the eight structures), between the re-docked and original crystallographic binding pose. The constraint settings are as follows: no constraints, 2 required out of 6 possible constraints, 3 required out of 6 possible constraints, 2 required out of 3 possible constraints (sec. amine anchors), 2 required out of 3 possible constraints (adenine core anchors).

A

Ligand	No constraint	2 of 6 required	3 of 6 required	2 of 3 (sidechain)	2 of 3 (adenine)
2(OH)-Pr-SNF	-12.897	-12.4	-12.423	-12.717	-12.845
3(OH)-Pr-SNF	-12.694	-8.923		-11.14	-11.468
Bn-SNF	-13.677	-10.897	-11.78	-11.095	-9.904
iBu-SNF	-12.710	-10.662	-8.668	-13.346	-12.004
Pe-SNF	-9.866	-10.448	-4.986	-10.616	-9.068
Pr-SNF	-12.672	-9.158	-9.505	-10.163	-12.637
SAH	-11.440	-9.701		-7.11	-11.327
SAM	-11.317	-7.562	-7.344	-7.728	-9.272
SNF	-11.624	-8.946	-6.729	-9.151	-11.463

B

Ligand	No constraint	2 of 6 required	3 of 6 required	2 of 3 (sidechain)	2 of 3 (adenine)
2(OH)-Pr-SNF	0.544	0.717	0.721	0.697	0.567
3(OH)-Pr-SNF	0.381	3.033		0.573	0.646
Bn-SNF	0.572	2.015	2.079	2.165	2.214
iBu-SNF	0.518	1.956	1.657	0.313	0.602
Pe-SNF	3.953	3.484	5.256	3.092	6.757
Pr-SNF	0.713	3.121	3.015	0.95	0.747
SAH	0.508	0.743		3.659	0.526
SAM	0.580	5.054	5.889	5.794	4.379
SNF	0.637	3.159	4.111	3.292	0.465

Next, the individual performance of the different docking grids was evaluated, and the constraint system described above was applied. Each of the nine ligands were redocked into each of the eight possible docking grids. Docking scores and RMSD values are reported in Table S5. From this data, we have come to the conclusion that by applying the combination of the receptor grids of the structures 5JLE[9] and 5LSY[1] (ensemble docking), along with the constraint requirements described earlier, the software was able to reproduce the crystallographic docking poses of the known ligands with satisfying docking scores and RMSD distances; this is summarized in Figure

S2A. Pe-SNF proved to be an outlier from this data, presumably because the relatively long n-pentyl chain in the molecule enables a large number of conformational states, significantly challenging the docking algorithm. Additionally, we have used single linkage clustering to assess whether these two protein structures sufficiently cover the conformational space of SETD2 binding pocket (Figure S2B).

Table S5. A) Docking scores for each ligand docked into each PDB structure. B) RMSD values for each ligand docked into each PDB structure (between the re-docked and original crystallographic binding pose).

A

Original structure	Ligand	5jle	5lss	5lsy	5lsz	4fmu	5lt6	5lt7	5lt8
5lt7	(2OH)-Pr-SNF	-9.707	-10.731	-12.030	-12.791	-10.857	-9.961	-12.897	-10.237
5lsz	(3OH)-Pr-SNF	-9.314	-9.299	-11.607	-12.694	-9.406	-6.430	-10.884	-8.840
5lsx	Bn-SNF	-8.300	-10.969	-13.677	-7.466	-10.926	-10.751	-13.357	-9.073
5lsy	iBu-SNF	-6.984	-11.319	-11.911	-12.710	-11.184	-9.893	-12.513	-8.641
5lt6	Pe-SNF	-8.105	-9.866	-8.444	-9.808	-8.999	-7.447	-9.602	-8.752
5lss	Pr-SNF	-8.760	-10.978	-11.603	-12.672	-8.705	-9.749	-9.125	-9.535
5jle	SAH	-11.440	-7.127	-10.766	-6.565	-7.396	-7.966	-7.506	-8.601
5v21	SAM	-11.317	-10.369	-9.899	-9.637	-8.041	-8.224	-8.203	-8.588
5lt8	SNF	-11.236	-11.170	-10.733	-11.624	-10.455	-6.193	-8.962	-9.066

B

Original structure	Ligand	5jle	5lss	5lsy	5lsz	4fmu	5lt6	5lt7	5lt8
5lt7	(2OH)-Pr-SNF	4.980	1.774	0.607	0.727	1.927	2.447	0.544	5.017
5lsz	(3OH)-Pr-SNF	4.895	3.962	0.832	0.381	3.965	7.546	1.922	4.767
5lsx	Bn-SNF	5.492	4.114	0.572	9.604	0.844	1.479	0.662	7.718
5lsy	iBu-SNF	6.764	1.673	0.533	0.846	1.806	2.903	0.659	5.019
5lt6	Pe-SNF	8.061	3.953	4.512	4.038	4.044	7.749	3.979	8.168
5lss	Pr-SNF	4.453	1.857	0.851	0.730	3.904	3.509	3.156	4.673
5jle	SAH	0.508	5.165	0.940	3.541	4.282	2.737	3.646	4.376
5v21	SAM	0.580	0.823	0.946	3.740	4.402	2.803	4.726	4.453
5lt8	SNF	0.492	0.687	0.816	0.637	0.831	8.401	4.088	4.110

Importantly, the docking protocol was further validated with the training set described earlier, also assessing the effect of applying the pharmacophore-based pre-screening. Briefly, the protocol retrieved all of the nine known actives with excellent early enrichment factors (ROC enrichments of 80 and 44 at 1% and 2% false positive rate, respectively[5]) and an area under the ROC curve (AUC) value of 0.97, which was further increased to 0.99 by the application of pharmacophore-based pre-screening. BEDROC values (at an α setting of 20.0) were 0.816 and 0.854 for these cases, respectively.

A

Grid	Ligand	Docking score	RMSD
5jle	SAM	-11.317	0.58
5lsy	SAH	-10.766	0.94
5lsy	Pr-SNF	-11.603	0.851
5lsy	Bn-SNF	-13.677	0.572
5lsy	iBu-SNF	-11.911	0.533
5lsy	(3OH)-Pr-SNF	-11.607	0.832
5lsy	Pe-SNF	-8.444	4.512
5lsy	(2OH)-Pr-SNF	-12.03	0.607
5jle	SNF	-11.236	0.492

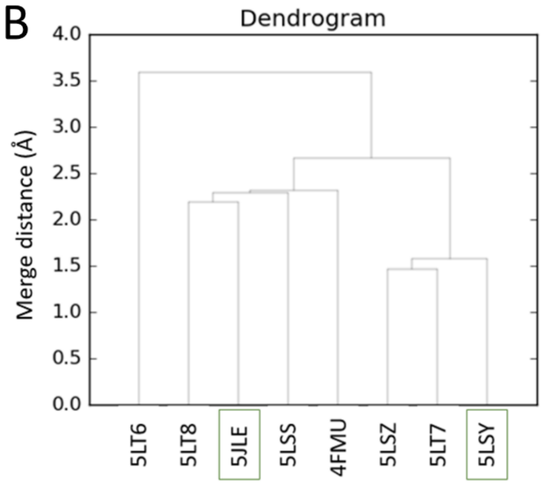

Figure S2. A) Docking results for the sinefungin analogs, using the combination of the 5JLE and 5LSY grids (ensemble docking). B) Single linkage clustering of the binding sites of the eight SETD2 structures. The structures 5JLE[9] and 5LSY[1] represent two clearly distinct sets of possible conformations. The structures 5LT6[1] is a singleton, which was not applied in the docking protocol due to its suboptimal performance (Table S4).

4. Substructure filter

With the aim of cutting down the screened dataset, while retaining compounds with at least a minimal structural resemblance to the natural cofactor SAM and sinefungin derivatives, a

substructure query was created, as summarized in Figure S3. In this general structure, A symbolizes any non-hydrogen atoms. The (a) notation on the terminal heavy atom means that the atom is in an aromatic bond, and the (X4) notation of the carbon connected to the basic nitrogen implies an sp^3 hybridization state. X is to be understood as either H or C(X4), allowing for (but not explicitly requiring) an arbitrary sidechain. The linker length is defined as $4 \leq n \leq 10$ (this allows for linear or ring-containing linkers as well). To summarize the requirements, we included compounds where a basic nitrogen atom (the importance of this functional unit was highlighted by Zheng et al.[2]) is found at a distance of 6-12 bonds from an aromatic ring (which mimics the adenine moiety in the known ligands).

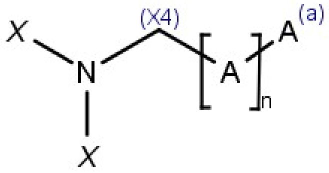

Figure S3. Substructure query

5. Virtual hits and primary screening results

Table S6. This table summarizes the structures, docking and IFP scores, primary screening results (SETD2 inhibition in an enzyme assay at 100 µM screening concentration), vendor availability and purity of the purchased virtual hit compounds. Purities are reported as normalized peak integrals from quality control LC-MS measurements, as reported by the respective vendors.

Name	MCULE ID	Structure	Docking score	IFP similarity	SETD2 inhibition @ 100µM (%)	Vendor	Vendor ID	Purity (%)

Name	MCULE ID	Structure	Docking score	IFP similarity	SETD2 inhibition @ 100µM (%)	Vendor	Vendor ID	Purity (%)
C1	MCULE-8470427732	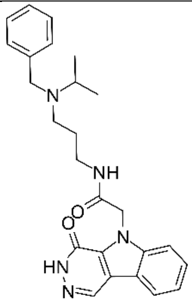	-11.284	0.518	Unavailable			
C2	MCULE-2766139226	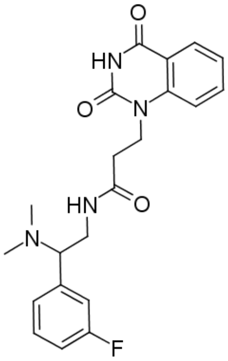	-12.347	0.308	69	Enamine	Z189630650	97
C2_2	MCULE-6642938474	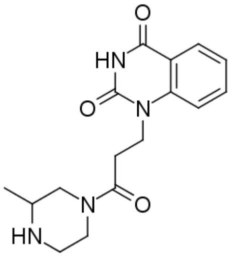	-11.736	0.450	68	Enamine	Z1457280314	95
C3	MCULE-4424512136	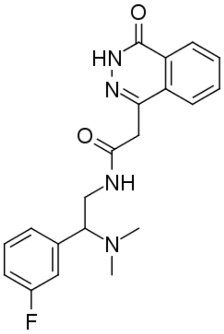	-11.554	0.330	n/a	Enamine	Z28553385	99+

Name	MCULE ID	Structure	Docking score	IFP similarity	SETD2 inhibition @ 100µM (%)	Vendor	Vendor ID	Purity (%)
C3_2	MCULE-4913493552	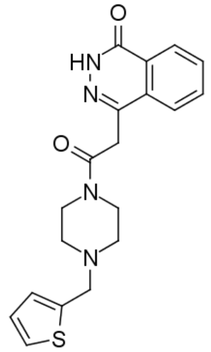	-11.230	0.313	76	Enamine	Z73429504	99+
C4	MCULE-3048299164	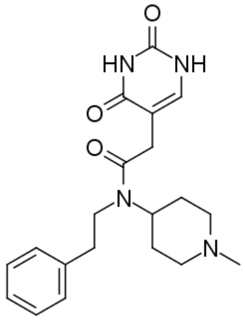	-10.351	0.278	62	Chembridge	78015614	99+
C5	MCULE-5532693412	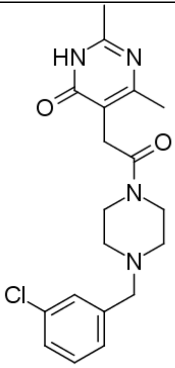	-10.940	0.309	27	Chembridge	37967057	99+

Name	MCULE ID	Structure	Docking score	IFP similarity	SETD2 inhibition @ 100µM (%)	Vendor	Vendor ID	Purity (%)
C6	MCULE-2122031183	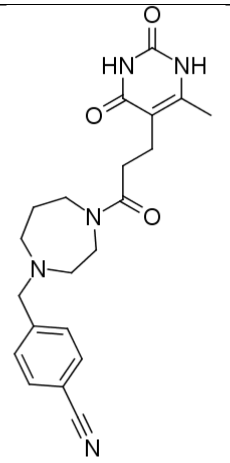	-11.475	0.461	58	Enamine	Z238004248	99+
C7	MCULE-8414174224	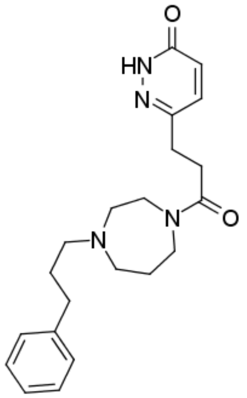	-11.423	0.412	37	Chembridge	65644214	99+
C8	MCULE-7362946334	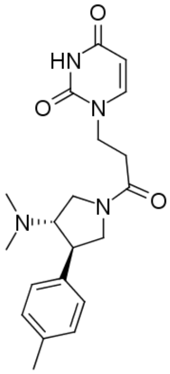	-10.448	0.309	46	Chembridge	18554635	99+

Name	MCULE ID	Structure	Docking score	IFP similarity	SETD2 inhibition @ 100µM (%)	Vendor	Vendor ID	Purity (%)
C9	MCULE-8896831552	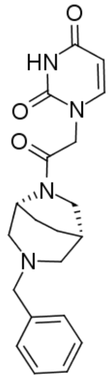	-11.604	0.281	82	Chembridge	75335369	99+
C9_2	MCULE-7693546359	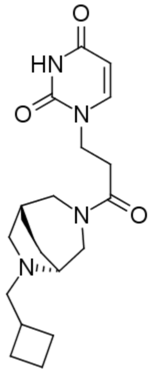	-10.396	0.289	54	Chembridge	31105034	99+
C9_3	MCULE-8975419856	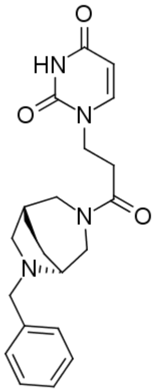	-11.240	0.327	n/a	Chembridge	16452556	99+

Name	MCULE ID	Structure	Docking score	IFP similarity	SETD2 inhibition @ 100μM (%)	Vendor	Vendor ID	Purity (%)
C10	MCULE-2814491647	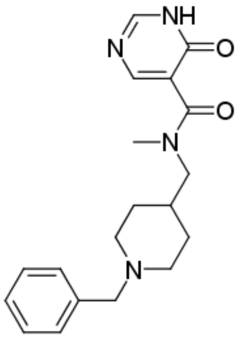	-10.825	0.309	64	Chembridge	64423528	89
C11	MCULE-3240644345	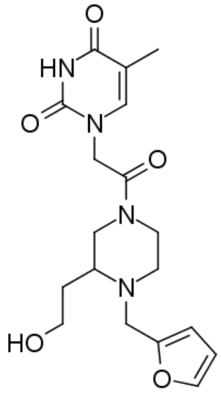	-11.623	0.362	91	Chembridge	96963852	92
C12	MCULE-1543772386	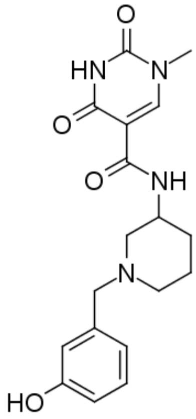	-12.093	0.274	n/a	Chembridge	30238080	99+

Name	MCULE ID	Structure	Docking score	IFP similarity	SETD2 inhibition @ 100µM (%)	Vendor	Vendor ID	Purity (%)
C12_2	MCULE-6638297500	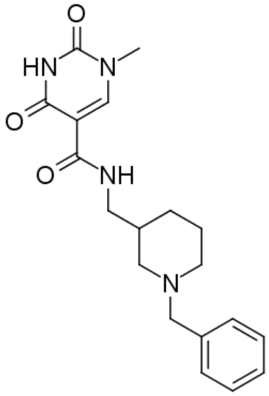	-11.373	0.305	n/a	Chembridge	84830096	99+
C13	MCULE-7341722870	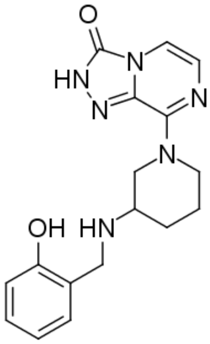	-12.435	0.354	83	Vitas	STL135578	99+
C14	MCULE-2483127491	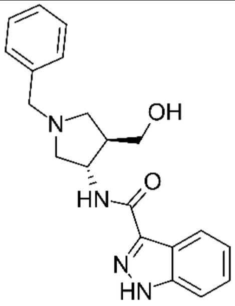	-10.714	0.312	Unavailable			

Name	MCULE ID	Structure	Docking score	IFP similarity	SETD2 inhibition @ 100μM (%)	Vendor	Vendor ID	Purity (%)
C15	MCULE-1531128187	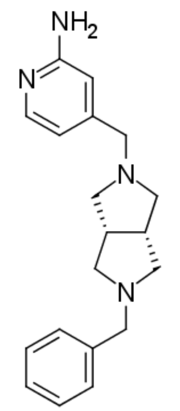	-10.288	0.309	n/a	Enamine	Z1939999448	99+
C16	MCULE-6546954086	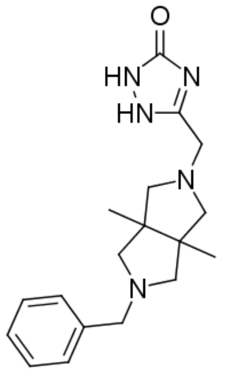	-11.358	0.351	19	Enamine	Z2734712913	97
C17	MCULE-7826477380	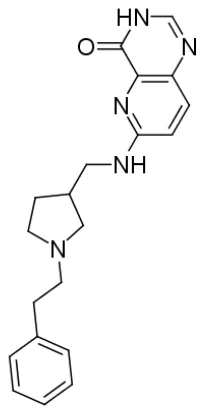	-11.308	0.290	69	Enamine	Z2582964869	91

Name	MCULE ID	Structure	Docking score	IFP similarity	SETD2 inhibition @ 100μM (%)	Vendor	Vendor ID	Purity (%)
C18	MCULE-9549108437	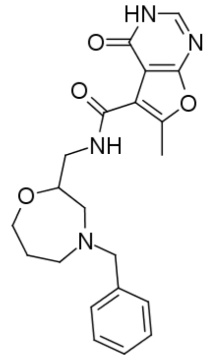	-11.279	0.223	73	Enamine	Z1866864777	99+
C19	MCULE-8052135474	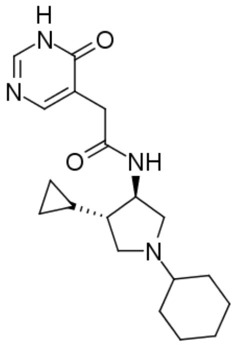	-10.410	0.298	80	Chembridge	90359892	94

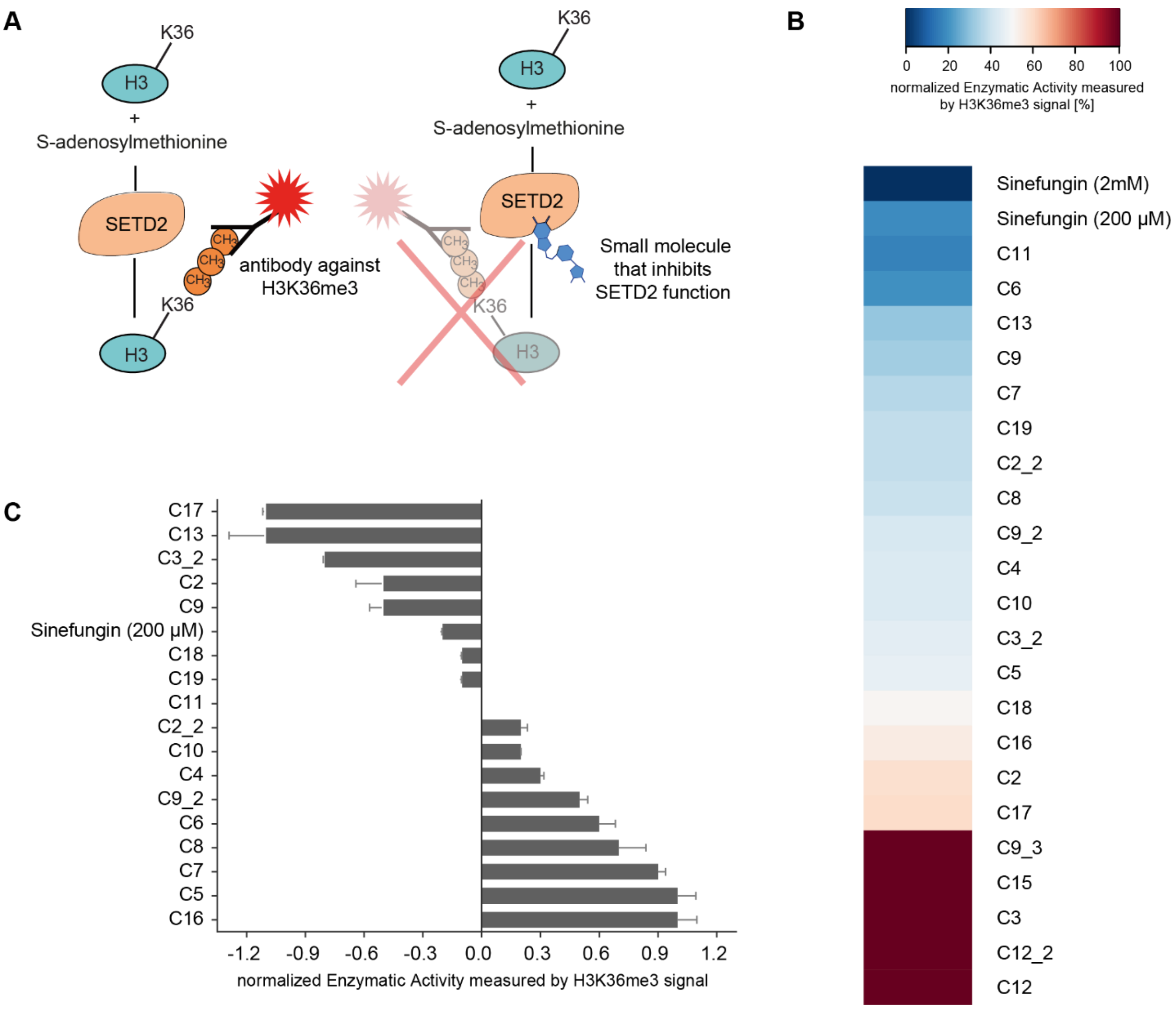

Figure S4. Screening of hit compounds with an enzymatic SETD2 assay A) Schematic representation of the chemiluminescence-based enzyme inhibition assay B) Heatmap of primary screening data of 22 virtual hits in singlets (100 µM). Values were blank corrected and normalized relative to positive control (full enzymatic activity) and to the negative control Sinefungin 2 mM (complete enzymatic activity inhibition). C) Secondary screen in duplicates at a concentration of 100 µM. Error bars depict the standard deviation [%]. Values were blank corrected and normalized.

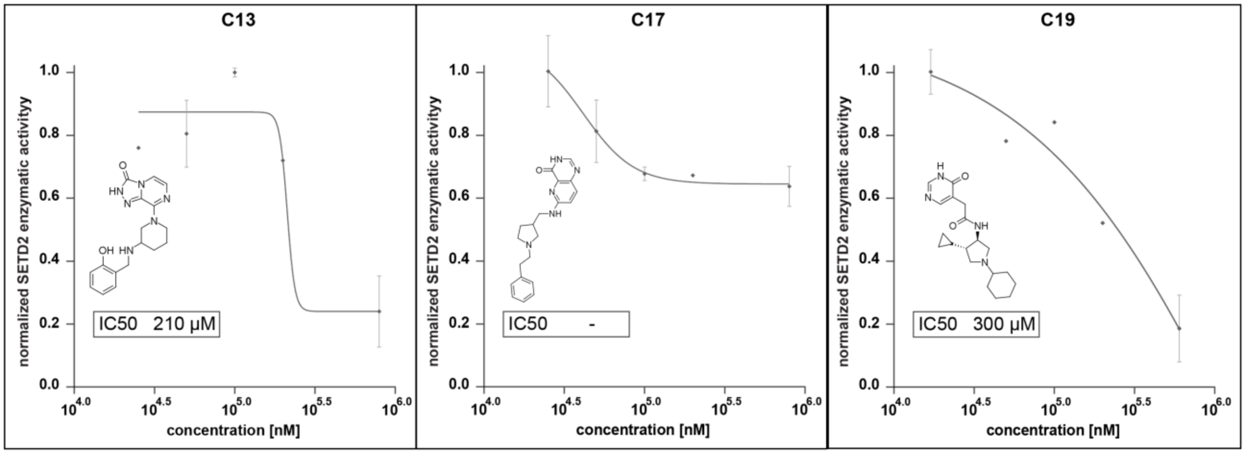

Figure S5. Enzymatic IC_{50} curves of the hit compounds C13 (A), C17 (B) and C19 (C).

6. Cellular Validation of hit compound C13

Cell culture

MOLM-13 and MV4-11 human leukemia cell lines were obtained from DSMZ (Deutsche Sammlung von Mikroorganismen und Zellkulturen GmbH, www.dsmz.de) and were cultured in RPMI 1640 (Gibco) supplemented with 10% FBS, 100 U/ml penicillin, 100 µg/ml streptomycin and 4 mM L-Glutamine.

For proliferation curves, cells were seeded at low densities ($4x10^5$ cells/mL) in biological triplicates and treated with C13 or DMSO, at indicated concentrations at 48 or 72 hours. Cell numbers were determined in regular intervals with the Intellicyt iQue Screener (Essen BioScience, Sartorius Group) and integrated with ForeCyte Software. The GraphPad Prism 8.0 software (San Diego, CA, USA) was used for statistical analyses. A two-tailed students t-test was used for p-value determination. * $p < 0.05$, ** $p < 0.01$, *** $p < 0.001$, **** $p < 0.0001$.

Western Blot

Western blotting was performed according to standard laboratory protocols. Briefly, cells were collected after 48 h of C13 treatment (100 µM), washed with PBS and whole-cell lysates were

prepared in Laemmli buffer. Samples were incubated at 95°C for 10 min, followed by 15 min of sonication. Protein concentrations were measured using the Pierce BCA Protein Assay Kit (Thermo scientific, 23225). Antibodies used were: anti-H3K36me3 (Abcam, 9050; 1:2000), anti-H3 (Abcam, 1791; 1:5000) and anti-H3K36me2 (Active Motif, 61019, 1:1000). Secondary antibodies used were: goat anti-mouse HRP (Thermo Fisher Scientific, 31430; 1:10000) and goat anti-rabbit HRP (Thermo Fisher Scientific 31460; 1:10000).

7. Other potential targets

Histone methyltransferase structures with a bound SAM, SAH or SNF ligand were collected from the Protein Data Bank and prepared with the default settings of Schrödinger Prime's One-step Protein Preparation interface. C13 was docked into these structures with the standard precision (SP) protocol of Glide, as reported in the main study. In Table S7, we have collected the Glide docking scores of the best binding pose of C13 in each of these structures and visually checked whether the core scaffold of C13 could mimic the binding mode of the adenine core of SAM/SAH/SNF (the latter was marked as "yes" if C13 reproduced 2 out of 2, or at least 2 out of 3 hydrogen bonds of the adenine core in the respective PDB structure). This analysis highlights six methyltransferases, where C13 was docked with a pose that i) has a docking score close to or below -10.0, and ii) reproduces the binding motif of the adenine core of the reference ligand. These are NSD1, ASH1L, SETMAR, EHMT2, SUV420H1 and SMYD2.

Table S7. Docking scores and visual check results of C13 in the binding pocket of other histone methyltransferase enzymes.

Name	Uniprot	PDB	Docking score	Core pose
NSD1	Q96L73	3OOI	-9.931	yes
		6KQP	-8.526	yes
NSD2 (WHSC1)	O96028	5LSU	-8.724	yes
ASH1L	Q9NR48	4YNM	-9.935	yes
SETD7	Q8WTS6	1N6A	-7.565	no
KMT2A (MLL1)	Q03164	2W5Y	-8.214	no
KMT2B (MLL2/MLL4)	Q9UMN6	7BRE	-7.958	no
KMT2D (MLL2/MLL4)	O14686	4Z4P	-9.408	no
KMT2C (MLL3)	Q8NEZ4	5F59	-7.966	no
SETMAR	Q53H47	3BO5	-10.069	yes
SUV39H2	Q9H5I1	2R3A	-8.343	yes
EHMT1 (GLP)	Q9H9B1	2IGQ	-9.357	yes
EHMT2 (G9a)	Q96KQ7	2O8J	-10.35	yes
SUV420H1	Q4FZB7	3S8P	-10.563	yes
SMYD2	Q9NRG4	3RIB	-10.718	yes
		3TG4	-11.321	yes
SMYD3	Q9H7B4	3MEK	-9.027	no
		3OXL	-9.254	no
		3OXG	-10.314	no
		3OXF	-9.449	yes
		3PDN	-7.903	no
		3RU0	-8.511	no

8. References

(1) Tisi, D.; Chiarparin, E.; Tamanini, E.; Pathuri, P.; Coyle, J. E.; Hold, A.; Holding, F. P.; Amin, N.; Martin, A. C. L.; Rich, S. J.; Berdini, V.; Yon, J.; Acklam, P.; Burke, R.; Drouin, L.; Harmer, J. E.; Jeganathan, F.; Van Montfort, R. L. M.; Newbatt, Y.; Tortorici, M.; Westlake, M.; Wood, A.; Hoelder, S.; Heightman, T. D. Structure of the Epigenetic Oncogene MMSET and Inhibition by N-Alkyl Sinefungin Derivatives. *ACS Chem. Biol.* **2016**, *11* (11), 3093–3105. https://doi.org/10.1021/acschembio.6b00308.

(2) Zheng, W.; Ibáñez, G.; Wu, H.; Blum, G.; Zeng, H.; Dong, A.; Li, F.; Hajian, T.; Allali-Hassani, A.; Amaya, M. F.; Siarheyeva, A.; Yu, W.; Brown, P. J.; Schapira, M.; Vedadi, M.; Min, J.; Luo, M. Sinefungin Derivatives as Inhibitors and Structure Probes of Protein Lysine Methyltransferase SETD2. *J. Am. Chem. Soc.* **2012**, *134* (43), 18004–18014. https://doi.org/10.1021/ja307060p.

(3) Salam, N. K.; Nuti, R.; Sherman, W. Novel Method for Generating Structure-Based Pharmacophores Using Energetic Analysis. *J. Chem. Inf. Model.* **2009**, *49* (10), 2356–2368. https://doi.org/10.1021/ci900212v.

(4) Loving, K.; Salam, N. K.; Sherman, W. Energetic Analysis of Fragment Docking and Application to Structure-Based Pharmacophore Hypothesis Generation. *J. Comput. Aided. Mol. Des.* **2009**, *23* (8), 541–554. https://doi.org/10.1007/s10822-009-9268-1.

(5) Nicholls, A. Confidence Limits, Error Bars and Method Comparison in Molecular Modeling. Part 1: The Calculation of Confidence Intervals. *J. Comput. Aided. Mol. Des.* **2014**, *28* (9), 887–918. https://doi.org/10.1007/s10822-014-9753-z.

(6) Schrödinger Release 2020-4: Schrödinger Suite 2020-4 Protein Preparation Wizard, LigPrep, Epik, ConfGen, Phase, Glide, Canvas; Schrödinger, LLC, New York, NY, 2020.

(7) Friesner, R. A.; Banks, J. L.; Murphy, R. B.; Halgren, T. A.; Klicic, J. J.; Mainz, D. T.; Repasky, M. P.; Knoll, E. H.; Shelley, M.; Perry, J. K.; Shaw, D. E.; Francis, P.; Shenkin, P. S. Glide: A New Approach for Rapid, Accurate Docking and Scoring. 1. Method and Assessment of Docking Accuracy. *J. Med. Chem.* **2004**, *47* (7), 1739–1749.

(8) Halgren, T. A.; Murphy, R. B.; Friesner, R. A.; Beard, H. S.; Frye, L. L.; Pollard, W. T.; Banks, J. L. Glide: A New Approach for Rapid, Accurate Docking and Scoring. 2. Enrichment Factors in Database Screening. *J. Med. Chem.* **2004**, *47* (7), 1750–1759.

(9) Yang, S.; Zheng, X.; Lu, C.; Li, G. M.; Allis, C. D.; Li, H. Molecular Basis for Oncohistone H3 Recognition by SETD2 Methyltransferase. *Genes Dev.* **2016**, *30* (14), 1611–1616. https://doi.org/10.1101/gad.284323.116.
